# Supplementary material for: Hyphal Als proteins act as CR3 ligands to promote immune responses against Candida albicans
Source: Nat Commun. 2024 May 9;15:3926. doi: 10.1038/s41467-024-48093-8 (PMC11082240; doi:10.1038/s41467-024-48093-8)
Supplement: Supplementary file 1 — Supplementary Information [file 41467_2024_48093_MOESM1_ESM.pdf]

Hyphal Als proteins act as CR3 ligands to promote immune responses against *Candida albicans*

Tingting Zhou[1], Norma V. Solis[2], Michaela Marshall[3], Qing Yao[4†], Rachel Garleb[1], Mengli Yang[1‡], Eric Pearlman[3], Scott G Filler[2,5], Haoping Liu[1*]

1 Department of Biological Chemistry, University of California, Irvine, CA, USA
2 Division of Infectious Diseases, Lundquist Institute for Biomedical Innovation at Harbor-UCLA Medical Center, Torrance, CA, USA
3 Department of Physiology and Biophysics, University of California, Irvine, CA, USA
4 Division of Biology and Biological Engineering, California Institute of Technology, Pasadena, CA, USA
5 David Geffen School of Medicine at UCLA, Los Angeles, CA, USA
* h4liu@uci.edu
Present address:
[†]Qing Yao. Gilead Sciences Inc. Foster City, CA, USA, 94404
[‡]Mengli Yang. Zymo Research Corporation. Irvine, CA, USA, 92614

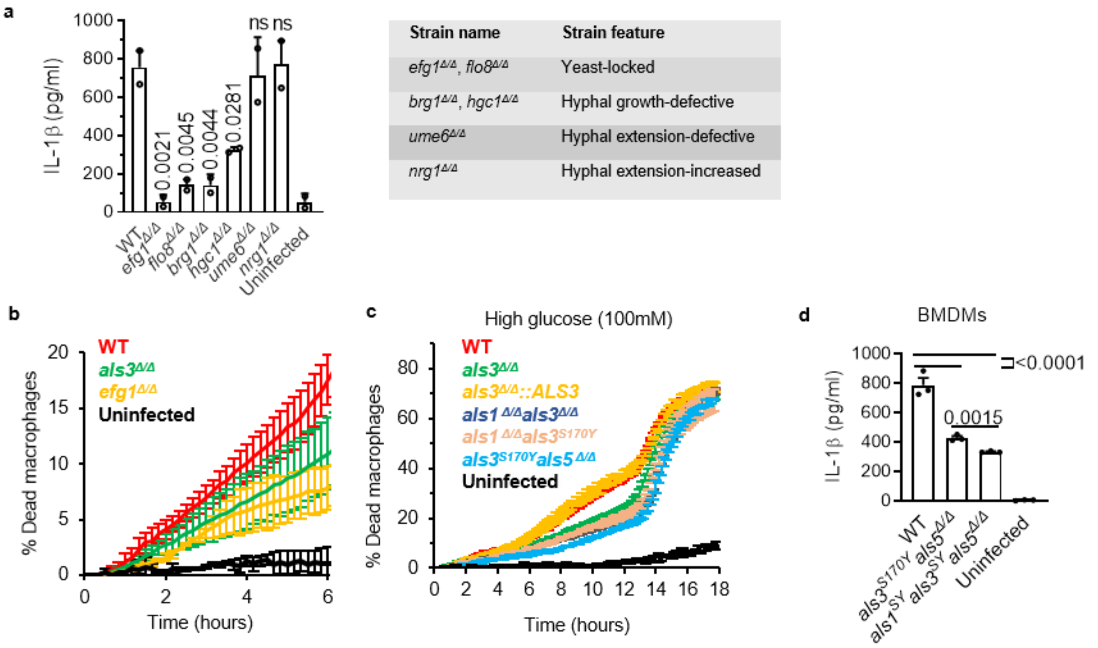

Supplementary Fig. 1 | The time course of Als3-mediated inflammasome activation.

a Production of IL-1β by J774A.1 cell line in response to *Candida albicans* transcription factor mutants. J774A.1 cells were primed by culturing for 2 h in a medium containing 50 ng ml^{-1} lipopolysaccharide (LPS). Subsequently, the *C. albicans* inoculum was added to microtiter plate wells at a multiplicity of infection of 1:1. After coculture for 5 h, the supernatants were removed, and the concentration of IL-1β was determined by enzyme-linked immunosorbent assay ELISA. The results were from the average values of two independent experiments (n=2). One-way ANOVA with Tukey post-hoc analysis was used for comparing strains with WT. **b**, **c** M1 macrophages were infected with the hyphal form of indicated *C. albicans* strains in Opti-MEM I medium (**b**) or Opti-MEM I medium with extra glucose (total 100 mM; **c**). Cell death was determined by kinetically measuring the uptake of Sytox Green. **d** BMDMs were stimulated with the hyphal form of *C. albicans* wild-type SN250, *als3*^{S170Y}*als5*$^{\Delta/\Delta}$ or *als1*^{SY}*3*^{SY}*5*$^{\Delta/\Delta}$ for 5.5 h. IL-1β secretion was determined by ELISA. One-way ANOVA with Tukey post-hoc analysis was used for comparing strains with SN250; unpaired two-tailed t-test analysis was used for comparing *als3*^{S170Y}*als5*$^{\Delta/\Delta}$ and *als1*^{SY}*als3*^{SY}*als5*$^{\Delta/\Delta}$ (n=3 from one representative experiment). The experiment was repeated at least three times with similar trends. Data in (**a, d**) are presented as mean ± SEM. Source data are provided as a Source Data file.

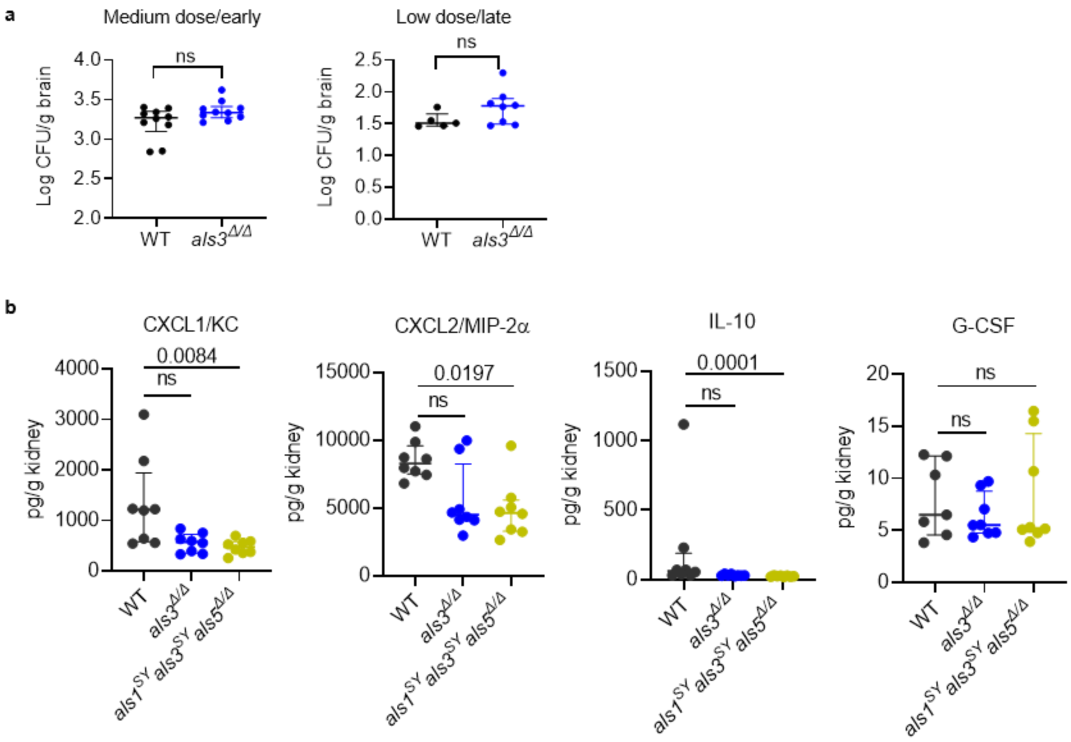

Supplementary Fig. 2 | Hyphal Als family protein promotes immune responses.

a The brain fungal burden of surviving mice after 18 hours (left, 10 mice) or 19 days of infection (right, 15 female mice infected totally; 5 mice survived after WT infection; 8 mice survived after the *als3*$^{\Delta/\Delta}$ mutant infection). Medium dose, $1x10^5$; low dose, $7.5x10^4$. *P* values were determined by two-tailed Mann-Whitney test. ns, not significant. **b** Level of indicated cytokines in kidneys after 2 days of infection. 8 female C57BL/6 mice were infected with each strain. *P* values were determined by Kruskal-Wallis test. ns, not significant. Results were median with interquartile range. Source data are provided as a Source Data file.

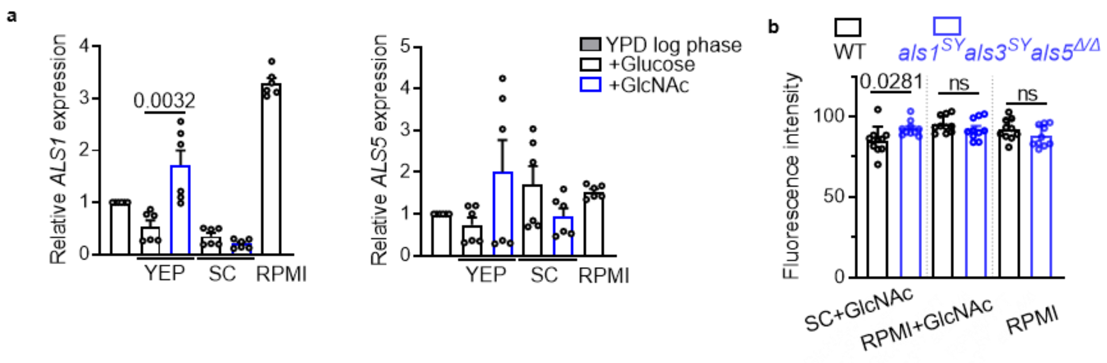

Supplementary Fig. 3 | Hyphal *ALS1* and *ALS5* gene expression analysis and β-glucan exposure analysis.

a Quantitative real-time RT-PCR was performed to assess *ALS1* and *ALS5* mRNA expression in SC5314 hyphae induced in the indicated medium. Expression levels were normalized to *ACT1* and set to 1 for log-phase SC5314 grown in YPD at 37°C. The data are presented as scatterplots, displaying individual values, and the mean ± SEM, derived from two independent experiments out of three conducted (n=6). Statistical analysis of the data from YEP media was performed using a two-tailed t-test. **b** Quantification of fluorescence intensity of *C. albicans* hyphae stained with Dectin-1-Fc and secondary antibody conjugated to FITC. Fluorescence intensities per area were quantitated by ImageJ (n=10). *P* values were determined by two-tailed t-test. Source data are provided as a Source Data file.

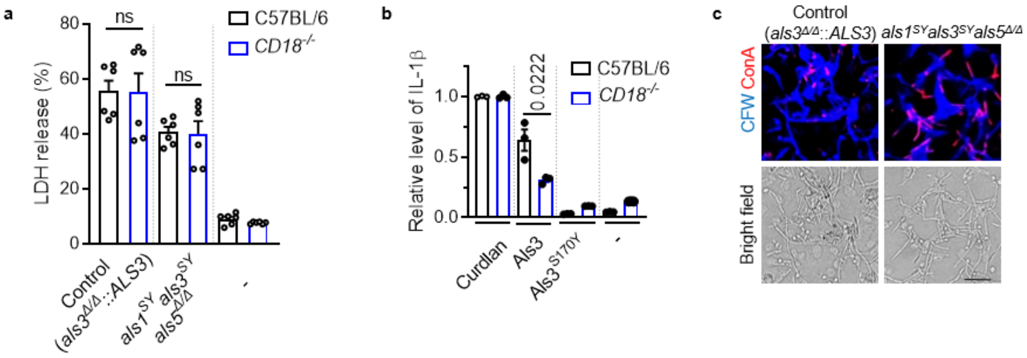

Supplementary Fig. 4 | CR3 is involved in Als3-mediated immune responses.
a BMDMs from C57BL/6 or *CD18*$^{-/-}$ mice were challenged with the hyphal form of *als3*$^{\Delta/\Delta}$*::ALS3* (control) or *als1*^{SY}*als3*^{SY}*als5*$^{\Delta/\Delta}$. Cell death was measured by LDH release after 6 hours. The data are pooled from two independent experiments (n=6). ns, not significant. **b** Purified His-tagged hyphal Als3 or Als3^{S170Y} protein was used to stimulate BMDCs for 24 h. The released IL-1β was measured by ELISA. Values were normalized to the average values of curdlan (100 µg/mL for 24 h)-stimulated BMDCs for each group (n=3 biological replicates). **c** Extracellular *C. albicans* hyphae at 2 hours post-infection were stained with concanavalin A- Alexa Fluor™ 633 Conjugate (red), and the total hyphae were stained with 10 µg/mL calcofluor white (Blue). Data in (**a, b**) are presented as mean ± SEM and were analyzed by a two-tailed t-test. Data in (**b, c**) are representative of three independent experiments. Source data are provided as a Source Data file.

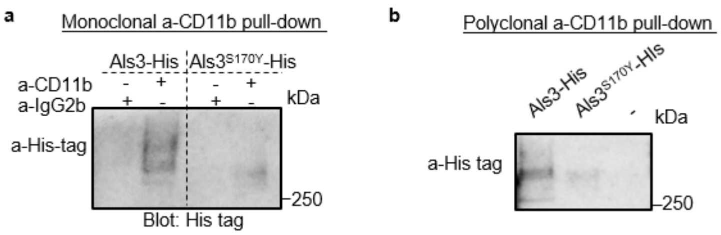

Supplementary Fig. 5 | Als3 interacts with CR3.

His-tagged Als3 or Als3^{S170Y} was mixed with iBMDM lysates. The mAb M1/70 (**a**) and a polyclonal antibody (**b**) were used to immunoprecipitate CD11b and its interacting proteins. Eluates were immunoblotted using an antibody against the His-tag. Data are representative of three independent experiments. Source data are provided as a Source Data file.

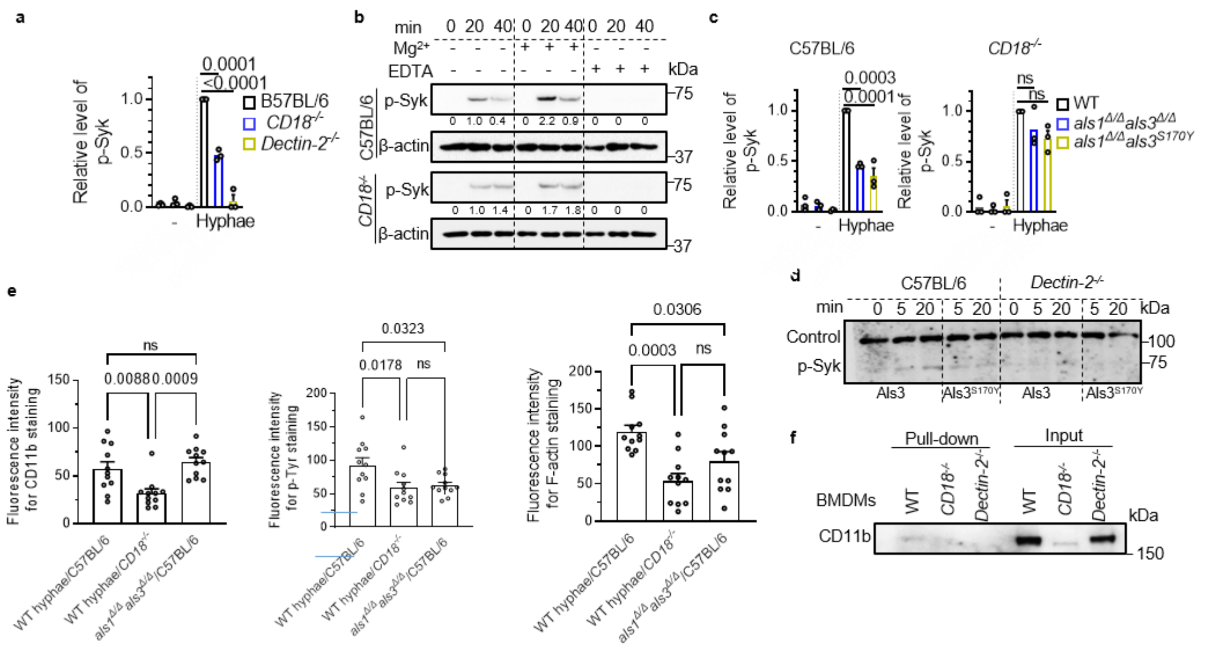

Supplementary Fig. 6 | Dectin-2 is essential for the "inside-out signaling" of CR3 in hyphal infection.

a Relative level of p-Syk in Fig. 6a at 20 min. $n = 3$ biologically independent samples. **b** Immunoblot analysis of p-Syk and β-actin from C57BL/6 and *CD18*$^{-/-}$ M0 BMDMs after infection with *C. albicans* hyphae with or without additional Mg^{2+} (additional 1 mM) or EDTA (5 mM). **c** Relative level of p-Syk in Fig. 6b at 20 min. $n = 3$ biologically independent samples. **d** Immunoblot analysis of p-Syk from C57BL/6 and *Dectin-2*$^{-/-}$ M0 BMDMs after incubation with 15 µg ml^{-1} Als3 or Als3^{S170Y} for indicated periods. **e** Quantification of the fluorescent intensity of immunostaining images for signal markers. 11 different areas for each condition were analyzed by using Leica LAS AF Lite software. **f** Activated CR3 pull-down assay for detecting the activated CR3 after *C. albicans* infection. The mAb clone CBRM1/5, which reacts with an activation-specific epitope of CR3, was used as a marker of the integrin conformational change to a high-affinity state[57, 77]. CBRM1/5-protein A beads were used to pull down active CR3 from the lysates of hypha-infected WT, *CD18*$^{-/-}$, or *Dectin-2*$^{-/-}$ BMDMs. *Elutes* were *immunoblotted* using a CD11b polyclonal antibody. Data are representative of three independent experiments. Data in (**a, c, e**) are presented as mean ± SEM. *P* values in (**a, c**) were determined by one-way ANOVA (Tukey's multiple comparisons test). ns, not significant. Source data are provided as a Source Data file.
