# Supplementary material for: Hyphal Als proteins act as CR3 ligands to promote immune responses against Candida albicans
Source: Nat Commun. 2024 May 9;15:3926. doi: 10.1038/s41467-024-48093-8 (PMC11082240; doi:10.1038/s41467-024-48093-8)
Supplement: Supplementary file 3 — Description of Additional Supplementary Files [file 41467_2024_48093_MOESM3_ESM.pdf]

Description of Additional Supplementary Files

File Name: Supplementary Data 1
Description: Strains and oligos used in this study.
