# Supplementary material for: Hyphal Als proteins act as CR3 ligands to promote immune responses against Candida albicans
Source: Nat Commun. 2024 May 9;15:3926. doi: 10.1038/s41467-024-48093-8 (PMC11082240; doi:10.1038/s41467-024-48093-8)
Supplement: Supplementary file 5 — Reporting Summary [file 41467_2024_48093_MOESM5_ESM.pdf]

nature portfolio

Corresponding author(s): Haoping Liu

Last updated by author(s): Mar 28, 2024

Reporting Summary

Nature Portfolio wishes to improve the reproducibility of the work that we publish. This form provides structure for consistency and transparency in reporting. For further information on Nature Portfolio policies, see our Editorial Policies and the Editorial Policy Checklist.

Statistics

For all statistical analyses, confirm that the following items are present in the figure legend, table legend, main text, or Methods section.

n/a	Confirmed	
☐	☒	The exact sample size (*n*) for each experimental group/condition, given as a discrete number and unit of measurement
☐	☒	A statement on whether measurements were taken from distinct samples or whether the same sample was measured repeatedly
☐	☒	The statistical test(s) used AND whether they are one- or two-sided<br>*Only common tests should be described solely by name; describe more complex techniques in the Methods section.*
☐	☒	A description of all covariates tested
☐	☒	A description of any assumptions or corrections, such as tests of normality and adjustment for multiple comparisons
☐	☒	A full description of the statistical parameters including central tendency (e.g. means) or other basic estimates (e.g. regression coefficient) AND variation (e.g. standard deviation) or associated estimates of uncertainty (e.g. confidence intervals)
☐	☒	For null hypothesis testing, the test statistic (e.g. *F*, *t*, *r*) with confidence intervals, effect sizes, degrees of freedom and *P* value noted<br>*Give P values as exact values whenever suitable.*
☒	☐	For Bayesian analysis, information on the choice of priors and Markov chain Monte Carlo settings
☒	☐	For hierarchical and complex designs, identification of the appropriate level for tests and full reporting of outcomes
☒	☐	Estimates of effect sizes (e.g. Cohen's *d*, Pearson's *r*), indicating how they were calculated

Our web collection on statistics for biologists contains articles on many of the points above.

Software and code

Policy information about availability of computer code

Data collection	qPCR was done by Bio-Rad CFX96 PCR System. Imaging was done by Leica SP8.
Data analysis	Imaging was analyzed with Image J (1.51j8) and Leica LAS AF software. GraphPad Prism software 9.5.1 was used for statistic analysis.

For manuscripts utilizing custom algorithms or software that are central to the research but not yet described in published literature, software must be made available to editors and reviewers. We strongly encourage code deposition in a community repository (e.g. GitHub). See the Nature Portfolio guidelines for submitting code & software for further information.

Data

Policy information about availability of data

All manuscripts must include a data availability statement. This statement should provide the following information, where applicable:

- Accession codes, unique identifiers, or web links for publicly available datasets
- A description of any restrictions on data availability
- For clinical datasets or third party data, please ensure that the statement adheres to our policy

The data supporting the findings from this study are available within the article file and its supplementary information/source data file. Any other raw data or noncommercial material used in this study are available from the corresponding author upon reasonable request. Source data are provided as a Source Data file. Source data are provided with this paper.

Research involving human participants, their data, or biological material

Policy information about studies with human participants or human data. See also policy information about sex, gender (identity/presentation), and sexual orientation and race, ethnicity and racism.

Reporting on sex and gender	No human participants or human data are used in this study.
Reporting on race, ethnicity, or other socially relevant groupings	No human participants or human data are used in this study.
Population characteristics	No human participants or human data are used in this study.
Recruitment	No human participants or human data are used in this study.
Ethics oversight	No human participants or human data are used in this study.

Note that full information on the approval of the study protocol must also be provided in the manuscript.

Field-specific reporting

Please select the one below that is the best fit for your research. If you are not sure, read the appropriate sections before making your selection.

☒ Life sciences ☐ Behavioural & social sciences ☐ Ecological, evolutionary & environmental sciences

For a reference copy of the document with all sections, see nature.com/documents/nr-reporting-summary-flat.pdf

Life sciences study design

All studies must disclose on these points even when the disclosure is negative.

Sample size	Statistical significance was determined using unpaired t-test, one-way ANOVA with Tukey's multiple comparisons test, two-tailed Mann-Whitney test, or Kruskal-Wallis test(GraphPad Prism). Differences were considered significant when the P value was <0.05.<br>The minimum of samples in each experiment was n=3 and up to n=15 except supplementary Fig 1.a which used two average values from two independent experiments. The exact n for each experiment was described in corresponding figure legends. Sample sizes were determined based on expected effect size and variability within the sample, previous experience and standards in the field. Low variability between the same type of samples, indicated as SME, confirming that n=3-6 samples is sufficient to observe statistically significant differences between relevant groups for most experiments. Whereas for fungal burden experiments, at least n=10 samples were used as figure legend indicated.
Data exclusions	No data was excluded from analysis.
Replication	At least two independent experiments were performed for each experiments, each panel presented the representative data or combined data. All reported data were reproduced reliably.
Randomization	For animal models, all mice used in this study were divided into different treatment groups randomly. For cell culture, cells were divided into each plates and assigned into different treatments groups randomly.
Blinding	All data acquisition and analysis in this study were performed in a blinded way.

Reporting for specific materials, systems and methods

We require information from authors about some types of materials, experimental systems and methods used in many studies. Here, indicate whether each material, system or method listed is relevant to your study. If you are not sure if a list item applies to your research, read the appropriate section before selecting a response.

Materials & experimental systems

n/a	Involved in the study	
☐	☒	Antibodies
☐	☒	Eukaryotic cell lines
☒	☐	Palaeontology and archaeology
☐	☒	Animals and other organisms
☒	☐	Clinical data
☒	☐	Dual use research of concern
☒	☐	Plants

Methods

n/a	Involved in the study	
☒	☐	ChIP-seq
☒	☐	Flow cytometry
☒	☐	MRI-based neuroimaging

Antibodies

Antibodies used

Antibody/ dilution/ company name/ catalog number/ clone number (monoclonal)/ Application
anti-IL-1β 1:2000 R&D Systems AF-401-NA N/A Western Blotting(WB)
anti-caspase-1 1:1000 AdipoGen AG-20B-0042--C100 Casper-1 WB
anti-GSDMD 1:1000 Abcam ab209845 EPR19828 WB
anti-β-actin-HRP 1:5000 CST 5125S 13E5 WB
anti-p-Syk 1:1000 CST 2710S C87C1 WB
anti-Syk 1:2000 CST 2712S N/A WB
anti-CD18 (Integrin β2) 1:1000 CST 47598S N/A WB
anti-p-p38 1:1000 CST 9211S N/A WB
anti-CD11b 1:1000 Novus NB110-89474 N/A WB
anti-his-HRP 1:2000 CST 9991S 27E8 WB
anti-rabbit-HRP 1:7500 Jackson Immuno Research Laboratories 111-035-003 N/A WB
anti-mouse-HRP 1:7500 Jackson Immuno Research Laboratories 115-035-003 N/A WB
anti-goat-HRP 1:7500 Jackson Immuno Research Laboratories 705-035-003 N/A WB

rabbit anti-p-Syk 1:100 CST 2710S C87C1 Immunofluorescence (IF)
mouse anti-pTyr 1:1600 CST 9411S P-Tyr-100 IF
rat anti-CD11b 1:100 BD Biosciences 553308 M1/70 IF
rat anti-Dectin-2 1:100 Bio-Rad MCA2415EL D2.11E4 IF
FITC-conjugated anti-mouse 1:100 Jackson ImmunoResearch 115-095-003 N/A IF
AlexaFluor488-conjugated anti-rabbit 1:100 Invitrogen A-11008 N/A IF
AlexaFluor568-conjugated anti-rat 1:100 Invitrogen A-11077 N/A IF
AlexaFluor647-conjugated anti-rat IgG2a 1:100 Biolegend 407511 N/A IF
eFluor 570-conjugated anti-rat IgG2b 1:100 Invitrogen 41-4815-82 N/A IF

ICRF44 2μg/reaction BioLegend 301302 ICRF44 Function
Mouse IgG1, κ 2μg/reaction BioLegend 400101 MOPC-21 Function
rat anti-CD11b 10μg/ml BD Biosciences 553308 M1/70 Function
Rat IgG2b, κ Isotype Control 10μg/ml Invitrogen 16-4031-81 eB149/10H5 Function

anti-CD11b 1:25 Invitrogen 14-0113-81 CBRM1/5 Immunoprecipitation (IP)
anti-CD11b 1:100 Novus NB110-89474 N/A IP
rat anti-CD11b 1:50 BD Biosciences 553308 M1/70 IP
Rat IgG2b, κ Isotype Control 1:100 Invitrogen 16-4031-81 eB149/10H5 IP

Validation

All antibodies were purchased from well-established and reputable companies.

Detailed validation from manufacturer's data sheets:
anti-IL-1β (# AF-401-NA; Recommended concentration 0.25 μg/mL, Validate for WB) https://www.rndsystems.com/products/mouse-il-1beta-il-1f2-antibody_af-401-na#product-details

anti-caspase-1(# AG-20B-0042--C100; Recommended concentration 1μg/ml, Validate for WB)
https://adipogen.com/ag-20b-0042-anti-caspase-1-p20-mouse-mab-casper-1.html

anti-GSDMD (# ab209845; Recommended concentration 1/1000, Validate for WB)
https://www.abcam.com/products/primary-antibodies/gsdmd-antibody-epr19828-ab209845.html

anti-β-actin-HRP (#5125S; Recommended concentration 1:1000, Validate for WB)
https://www.cellsignal.com/products/antibody-conjugates/b-actin-13e5-rabbit-mab-hrp-conjugate/5125

anti-p-Syk (#5125S; Recommended concentration 1:1000, Validate for WB; Recommended concentration 1:100-1:400, Validate for IF)
https://www.cellsignal.com/products/primary-antibodies/phospho-syk-tyr525-526-c87c1-rabbit-mab/2710

anti-Syk (#2712; Recommended concentration 1:1000, Validate for WB)
https://www.cellsignal.com/products/primary-antibodies/syk-antibody/2712

anti-CD18 (Integrin β2) (#47598S; Recommended concentration 1:1000, Validate for WB)
https://www.cellsignal.com/products/primary-antibodies/integrin-b2-antibody/47598

anti-p-p38 (#9211S; Recommended concentration 1:1000, Validate for WB)
https://www.cellsignal.com/products/primary-antibodies/phospho-p38-mapk-thr180-tyr182-antibody/9211

anti-CD11b (#NB110-89474; Recommended concentration 2 μg/ml, Validate for WB)
https://www.novusbio.com/products/cd11b-antibody_nb110-89474

anti-his-HRP (#9991S; Recommended concentration 1:1000, Validate for WB)
https://www.cellsignal.com/products/antibody-conjugates/his-tag-27e8-mouse-mab-hrp-conjugate/9991

anti-rabbit-HRP (#111-035-003; Recommended concentration 1:10,000 - 1:200,000 for WB)
https://www.jacksonimmuno.com/catalog/products/111-035-003

anti-mouse-HRP (#115-035-003; Recommended concentration 1:10,000 - 1:200,000 for WB)
https://www.jacksonimmuno.com/catalog/products/115-035-003

anti-goat-HRP (#705-035-003; Recommended concentration 1:10,000 - 1:200,000 for WB)
https://www.jacksonimmuno.com/catalog/products/705-035-003

mouse anti-pTyr (#9411S; Recommended concentration 1:1600 - 1:3200, Validate for Immunofluorescence)
https://www.cellsignal.com/products/primary-antibodies/phospho-tyrosine-mouse-mab-p-tyr-100/9411

rat anti-CD11b (#553308)
Validated application including Blocking, Immunohistochemistry-frozen, Immunoprecipitation (Reported). https://www.bdbiosciences.com/en-eu/products/reagents/flow-cytometry-reagents/research-reagents/single-color-antibodies-ruo/purified-rat-anti-cd11b.553308
Validated application for IF in reference papers, for example PMID: 34001904.

rat anti-Dectin-2 (#MCA2415EL)
Validated application including flow cytometry, immunohistology – frozen, and immunoprecipitation
https://www.bio-rad-antibodies.com/monoclonal/mouse-dectin-2-antibody-d2-11e4-mca2415.html?f=purified

FITC-conjugated anti-mouse (#115-095-003; Recommended concentration 1:50 - 1:200, validate for IF)
https://www.jacksonimmuno.com/catalog/products/115-095-003

AlexaFluor488-conjugated anti-rabbit (#A-11008; Recommended concentration 4 µg/mL, validate for Immunocytochemistry (ICC/IF))
https://www.thermofisher.com/antibody/product/Goat-anti-Rabbit-IgG-H-L-Cross-Adsorbed-Secondary-Antibody-Polyclonal/A-11008

AlexaFluor568-conjugated anti-rat (#A-11077; Recommended concentration 1-10 µg/mL, validate for Immunocytochemistry (ICC/IF))
https://www.thermofisher.com/antibody/product/Goat-anti-Rat-IgG-H-L-Cross-Adsorbed-Secondary-Antibody-Polyclonal/A-11077

AlexaFluor647-conjugated anti-rat IgG2a (#407511)
References: PMID: 34348160, Validate for immunostaining
https://www.biolegend.com/fr-ch/products/alexa-fluor-647-anti-rat-igg2a-antibody-14984
eFluor 570-conjugated anti-rat IgG2b (#41-4815-82)
https://www.thermofisher.com/antibody/product/Mouse-anti-Rat-IgG2b-Secondary-Antibody-clone-R2B-7C3-Monoclonal/41-4815-82
ICRF44 (#301302; I-domain binding in reference PMID: 26650353)
https://www.biolegend.com/de-de/products/purified-anti-human-cd11b-antibody-770?GroupID=BLG9916
Mouse IgG1, κ (#400101; Validate for functional assay)
https://www.biolegend.com/de-de/products/purified-mouse-igg1-kappa-isotype-ctrl-1375
Rat IgG2b, κ Isotype Control (#16-4031-81; Validate for functional assay)
https://www.thermofisher.com/antibody/product/Rat-IgG2b-kappa-clone-eB149-10H5-Isotype-Control/16-4031-81
anti-CD11b (#14-0113-81)
Functional assay references: PMID: 19454663; PMID: 18768756.
Functional assay reference for mouse BMDM: PMID: 19666536.
https://www.thermofisher.com/antibody/product/CD11b-activation-epitope-Antibody-clone-CBRM1-5-Monoclonal/14-0113-81

Eukaryotic cell lines

Policy information about cell lines and Sex and Gender in Research

Cell line source(s)	iBMDM from Dr. Katherine A. Fitzgerald
Authentication	No authentication. But It has been confirmed in lab by testing some macrophage responses, for example pyroptosis with LPS +ATP.
Mycoplasma contamination	It's regularly tested in lab and confirmed to be free of contamination.
Commonly misidentified lines (See ICLAC register)	*Name any commonly misidentified cell lines used in the study and provide a rationale for their use.*

Animals and other research organisms

Policy information about studies involving animals; ARRIVE guidelines recommended for reporting animal research, and Sex and Gender in Research

Laboratory animals	CD18–/– mice were originally provided by Claire Doerschuk (University of North Carolina, Chapel Hill, NC, USA). Dectin-2-deficient mice were a kind gift from Yoichiro Iwakura (University of Tokyo, Tokyo, Japan). Age- and sex-matched C57BL/6J mice were purchased from Jackson Laboratories (Bar Harbor, ME, USA). 6-week-old male and female C57BL/6J mice were purchased for in vivo experiments. All animals were bred in pathogen-free conditions in microisolator cages and were treated according to institutional guidelines following approval by the University of California IACUC. Male and female mice at the age of 6-12 weeks were used for the

	generation of BMDMs and BMDCs.
Wild animals	No wild animals are used in this study
Reporting on sex	Both male and female mice were used in the experiments, and we found no difference between them.
Field-collected samples	No filed-collected samples are used in this study
Ethics oversight	All animals for in vitro work were bred in pathogen-free conditions in microisolator cages and were treated according to institutional guidelines following approval by the University of California, Irvine IACUC. All animal in vivo work was approved by the Institutional Animal Care and Use Committee at the Lundquist Institute for Biomedical Innovation at Harbor-UCLA Medical Center.

Note that full information on the approval of the study protocol must also be provided in the manuscript.

Plants

Seed stocks	No plants are used in this study.
Novel plant genotypes	No plants are used in this study.
Authentication	No plants are used in this study.
